# Genome-wide association studies revealed partial genetic links between early vigour and precocity in macadamia

**DOI:** 10.1093/hr/uhaf162

**Published:** 2025-07-04

**Authors:** Pragya Dhakal Poudel, Joanne-De Faveri, Bruce Topp, Mobashwer Alam

**Affiliations:** Queensland Alliance for Agriculture and Food Innovation, The University of Queensland, St Lucia, QLD 4072, Australia; AV Data Analytics, Adelaide, SA 5153, Australia; Queensland Alliance for Agriculture and Food Innovation, The University of Queensland, St Lucia, QLD 4072, Australia; Queensland Alliance for Agriculture and Food Innovation, The University of Queensland, St Lucia, QLD 4072, Australia

## Abstract

Early vigour (EV) and precocity are important traits for orchard establishment and profitability in macadamia. EV determines tree growth and adaptation, while precocity facilitates early yield, offering economic benefits. Although, a positive relationship between these traits has been observed in other tree crops, their association in macadamia remains unclear. This study aimed to identify genetic links between EV and precocity by assessing genetic variability, heritability, and correlations in a 5-year-old macadamia breeding population. The population comprised 904 progenies planted across six sites in Queensland, Australia. Genome-wide association studies (GWAS) were conducted on a subset of 220 accessions genotyped with 7401 SNP markers. A linear mixed model incorporating a kinship matrix and principal components to account for population structure was used to perform association analysis in TASSEL. Phenotypic analyses in ASReml-R revealed that precocity had higher broad- (H^2^ = 0.25–0.84) and narrow-sense (h^2^ = 0.10–0.77) heritability compared to EV (H^2^ = 0–0.61, h^2^ = 0–0.49). EV and precocity showed positive phenotypic (0.25–0.42) and genetic (0.21–0.31) correlations. GWAS identified 11 significant markers (false discovery rate < 0.05), including two pleiotropic markers (*Mint10079* and *Mint4004*) associated with both EV and precocity. Putative genes linked to these markers were involved in cell wall modelling, pathogen defence, abiotic stress tolerance, flowering, overall growth, and development in other tree species. These significant markers, postvalidation, hold substantial promise for utilization in marker-assisted selection (MAS). Integrating putative pleiotropic markers into MAS can enhance genetic gain by reducing the selection time for and enabling simultaneous selection for EV and precocity.

## Introduction

Macadamia (*Macadamia integrifolia, Macadamia ternifolia*, and hybrids) is a native Australian tree crop cultivated globally for its high-value kernels. Australia is one of the leading producers with cultivation expanding into subtropical and tropical regions around the world, including China, South Africa, Hawaii, New Zealand, South America, and Southeast Asia [1]. Despite its popularity, the genetic improvement of macadamia has been slow due to obstacles such as high heterozygosity, protracted juvenility, and long breeding cycle ([2–4]). Genetic gains are further impeded by vigourous vegetative growth at maturity, which restrict orchards to low-density planting with high management costs [5, 6]. Traditional breeding approaches involve laborious phenotyping over many years to identify elite candidate cultivars, hence it takes at least 24 years to release new varieties [1]. Industry may thus benefit from genomic approaches that reduce selection cycles and increase genetic gain.

Macadamia trees remain juvenile for at least 5–7 years until maturing at 10–15 years [1]. Long juvenility extends the breeding cycle, slowing the development of new cultivars, while growers face the challenge of delayed orchard productivity, requiring substantial financial investments without returns for several years. Precocity refers to the ability of a tree crop to bear fruits or flowers early in its life cycle [7]. It is associated with a short juvenile period and high productivity at maturity in wood crops (Zimmerman, 1972 [127]). In macadamia, precocity is particularly valuable as it accelerates breeding progress by reducing selection cycles and increasing genetic gain [8]. Maximizing early yields and shortening the time to first commercial harvest are top priorities for growers when establishing new macadamia orchards [9]. Precocity offers the benefit of early productivity and faster economic returns for growers. In the absence of genetically precocious cultivars, methods such as trunk girdling and application of plant growth regulators have shown to increase early fruiting [10]. Developing naturally precocious cultivars would optimize both breeding efficiency and orchard productivity, reducing reliance on management interventions.

Early vigour (EV) or growth of young trees is another important trait with clear advantages in tree crops, although vigour at maturity is considered detrimental for productivity [11]. EV determines crop’s ability to cope with biotic and abiotic stresses with significant implications on climate change adaptation such as drought and heat [12]. For instance, increased early biomass promotes rapid canopy closure, reducing soil evaporation and improving water availability [13]. High early shoot growth also facilitates better photosynthate transport to roots, ensuring proper establishment and resilience against stress. This has been observed in crops like cotton, wheat, rice, *Arabidopsis,* and some forest trees [12, 14, 15]. Furthermore, stem diameter in young plants correlates with traits associated with water absorption and transport, supporting survival during water stress [16]. In macadamia, EV is particularly important for establishing a healthy and uniform orchard canopy, which enhances a plant’s ability to compete for resources like sunlight, water, nutrients, and contributes to a greater crop output.

Strong EV is also linked to precocity because it is essential to reach an appropriate growth size in order to enter the reproductive phase [17]. EV expedites the transition from juvenility to the adult phase, a process marked by flowering. The positive relationship between EV and precocity has been well established in several tree crops such as apple, pear, pecan, cocoa, and olive [17–20]. Increased light interception had been suggested as a contributing factor to this relationship [21]. Since EV accelerates canopy closure, it optimizes light interception and supports precocity [22]. For instance, growth of 1- to 4-year-old pecan seedlings showed positive correlations with precocity, with the highest correlations observed in later growth stages [17]. In addition, establishment of this EV and precocity relationship has allowed preselection of precocious cultivars on the basis of early vegetative growth in tree crops [18–20, 23]. However, in macadamia, the only study reporting positive influence of growth on yield in 4 to 6-year-old trees was limited to 15 genotypes [21], with no explorations into the genetic basis of this relationship. Exploring such genetic links would facilitate the selection of one trait based on the other, thereby reducing evaluation time and enhancing genetic gain.

Correlated traits often share genes or quantitative trait loci (QTL/s) that are located in proximate genomic regions [24]. Understanding the genetic basis of traits also facilitates the selection of new candidate cultivars. One of the most useful methods of dissecting the genetic components of complex quantitative traits is through genome-wide association studies (GWAS). GWAS is a genomic approach to identify candidate gene/QTL associated with target trait [25]. Significant marker–trait associations from GWAS facilitate marker-assisted selection (MAS), expediting breeding by enabling the selection for desirable traits during the juvenile stage, hence eliminating the necessity for field evaluations to maturity [25].

However, individual GWAS studies on the same trait often yield inconsistent results due to factors such as population-specific allele frequencies, inadequate control of population structure, and environmental influences [26]. Additionally, GWAS findings require validation across multiple environments to ensure their reliability [27]. A promising approach to address these limitations is meta-analysis of GWAS (metaGWAS), which integrates summary statistics (e.g. marker effects and their standard errors) from multiple independent studies to increase statistical power and reduce false-positive associations [28]. By combining data from different populations and environments, metaGWAS provides more robust and generalizable insights into the genetic architecture of traits. Despite its advantages, meta-analysis of QTLs has been extensively applied in grain crops [29–31], but remains rare in fruit and nut crops. The limited number of GWAS in these species poses a challenge for conducting metaGWAS, as this approach relies on multiple independent studies to combine data effectively. In macadamia, metaGWAS is currently not feasible due to the scarcity of GWAS studies. While recent efforts have identified marker–trait associations for yield and nut-related traits [32–34], traits such as EV and precocity remain unexplored.

To address this gap, this study aims to investigate the genetic basis of EV and precocity in macadamia through GWAS. Specifically, this study measured the genetic variance and heritability of EV and precocity in seedling progenies and estimated phenotypic and genetic correlations among examined traits. A GWAS was performed to identify candidate genes associated with EV and precocity traits and common genetic mechanism of traits were investigated. Results of this study provide the genetic links of EV and precocity and highlight the potential of early and rapid selection for these traits in future breeding programmes.

## Results

### Phenotypic variation

A summary of the mean and standard errors (SEs) of the raw data revealed a range of phenotypic variation across sites for all traits (Table 1). The highest means for EV traits were observed at Dunoon, where height (HT), canopy width (CW), and canopy volume (CV) were 4.94 m, 3.77 m, and 85.42 m^3^, respectively. The lowest means for HT (3.27 m) and CV (31.28 m^3^) were recorded at Newrybar, while the lowest mean for CW (2.67 m) was found at Baffle Creek_S1. Similarly, precocity traits, as represented by total nut-in-shell mass (TNM) and cumulative nut-in-shell mass (CNM), were lowest at Baffle Creek_S1, with mean values of 474.8 and 478.4 g, respectively. In contrast, the highest means for TNM (4181 g) and CNM (6563 g) were observed at Bundaberg.

**Table 1:** Mean, maximum (max), and minimum (min) of EV and precocity traits across sites

Traits	Description	Alloway	Ammamoor_S1	Ammamoor_S2	Baffle Creek_S1	Baffle Creek_S2	Bundaberg	Dunoon	Newrybar
Height (m)	Mean (SE)	3.77 (0.04)	3.93 (0.05)	4.25 (0.06)	3.88 (0.1)	4.8 (0.06)	4 (0.09)	4.94 (0.13)	3.27 (0.06)
	Max	5.02	5.47	5.80	6.01	6.86	6.23	7.8	4.88
	Min	1.16	1.52	2.6	1.5	1.3	1.99	1.9	1.28
Canopy width (m)	Mean (SE)	3.43 (0.05)	2.71 (0.05)	3.25 (0.06)	2.67 (0.1)	3.8 (0.07)	3.21 (0.09)	3.77 (0.11)	2.83 (0.06)
	Max	4.53	4.27	5.00	4.45	5.90	5.20	5.80	4.85
	Min	0.79	0.84	1.4	0.73	0.8	1.2	1	0.84
Canopy volume (m^3^)	Mean (SE)	49.12 (1.38)	33.55 (1.34)	50.54 (2.23)	34.31 (2.68)	81.51 (3.31)	48.18 (3.27)	85.42 (5.42)	31.28 (1.66)
	Max	94.98	99.71	120.74	114.80	220.77	133.71	247.18	105.72
	Min	0.76	1.44	7.60	0.86	1.36	4.04	1.99	0.95
Total nut in shell mass (g)	Mean (SE)	2297 (175)	1170 (135)	705.2 (84)	474.8 (129)	2892 (243)	4181 (358)	1535 (273)	888.9 (128)
	Max	8997	8388	4666	4960	17 984	12 159	11 400	6714
	Min	0	0	0	0	0	0	0	0
Cumulative nut in shell mass (g)	Mean (SE)	2297 (175)	1288 (155)	1401 (170)	478.4 (129)	3564 (310)	6563 (578)	1734 (326)	940.6 (136)
	Max	8997	9600	8541	4960	22 114	18 661	15 630	7428
	Min	0	0	0	0	0	0	0	0

### Genetic variance and heritability

The multisite analysis revealed significant differences (*P* < 0.05, Walds test, Supplementary Table 1) among sites for all traits. Substantial differences in additive genetic and residual variances for each trait across sites were observed (Table 2, Supplementary Table 2). Heritability estimates varied widely among sites and traits. Broad-sense (H^2^) and narrow-sense heritability (h^2^) values were generally higher for precocity traits compared to EV traits. For precocity traits, the average h^2^ and H^2^ were 0.27 and 0.48 for CNM, and 0.28 and 0.50 for TNM, respectively. Among EV traits, HT showed the highest heritability, with h^2^ averaging 0.22 and H^2^ at 0.41. In contrast, CW had the lowest heritability estimates, with an average h^2^ of 0.09 and H^2^ of 0.21. CV exhibited moderate heritability, with h^2^ averaging 0.12 and H^2^ at 0.23.

**Table 2:** Range of variance components, heritability, and average heritability across sites for EV and precocity traits. Detailed numeric data on variance components and heritability of each trait at each site is presented in Supplementary Table 2

Trait	V_a_ (% of V_p_)	V_f_ (% of V_p_)	V_e_ (% of V_p_)	h^2^	H^2^	Average h^2^	Average H^2^
HT	0%–49%	0%–44%	39%–72%	0–0.49	0.28–0.61	0.22	0.41
CW	0%–36%	0%–28%	57%–100%	0–0.36	0–0.42	0.09	0.21
CV	0%–35%	0%–18%	54%–100%	0–0.34	0–0.46	0.12	0.23
TNM	10%–77%	0%–62%	16%–75%	0.10–0.77	0.25–0.84	0.28	0.50
CNM	10%–70%	9%–62%	16%–75%	0.12–0.70	0.25–0.84	0.27	0.48

V_a_, additive genetic variance; V_p_, total phenotypic variance; V_f_, family variance; V_e_, residual variance; h^2^, narrow-sense heritability; H^2^, broad-sense heritability

### Phenotypic and genetic correlations

Phenotypic correlations among EV and precocity traits were positive and significant (r_p_ = 0.25–0.42, *P* < 0.001; Table 3). There was also some degree of correlation between Best Linear Unbiased Predictions (BLUPs) (r_g_ = 0.21–0.31) among these traits. Highest r_p_ and r_g_ for EV and precocity were observed for the pair CW-TNM and the lowest for HT-CNM.

**Table 3:** Phenotypic correlations (top right, r_p_) and correlation between BLUPs (bottom left, r_g_) between EV and precocity traits

r_p_ r_g_	HT	CW	CV	TNM	CNM
				
HT		0.66^***^	0.78^***^	0.28^***^	0.27^***^
CW	0.64^***^		0.91^***^	0.42^***^	0.39^***^
CV	0.78^***^	0.94^***^		0.39^***^	0.36^***^
TNM	0.29^***^	0.28^***^	0.31^***^		0.96^***^
CNM	0.21^***^	0.22^***^	0.23^***^	0.94^***^	

‘*’ in the superscript represents the level of significance of the correlation component (***P < 0.001).

### Genome-wide associations

**Figure 1:**
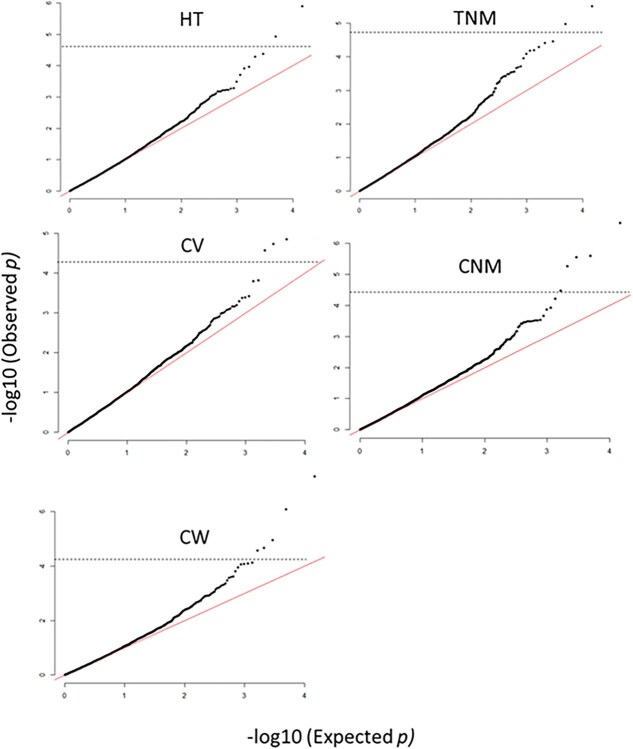
QQ plots showing expected versus observed significant levels for EV and precocity traits. Each black dot represents one of the total SNP markers in the study. Red diagonal lines indicate the expected *P-*values. SNP markers above the dashed horizontal lines (FDR = 0.05) are significantly associated with the trait.

**Figure 2:**
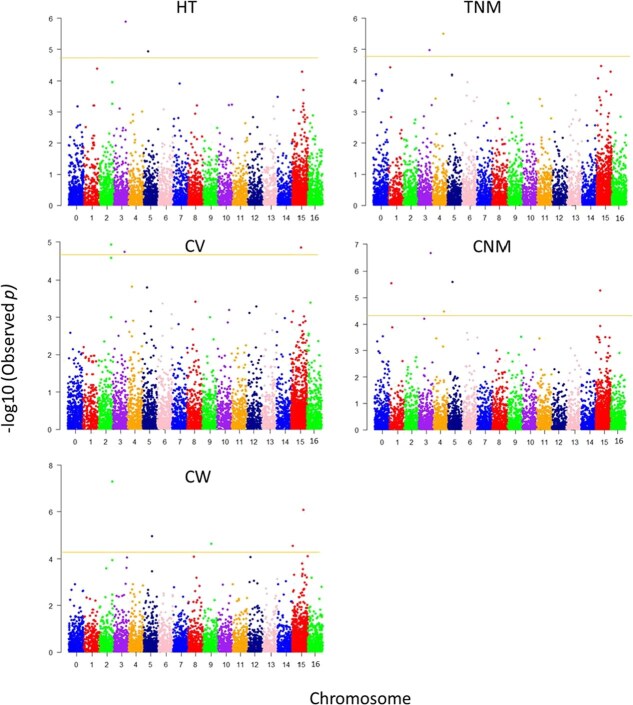
Manhattan plots showing distribution of SNPs across the macadamia genome and significance (−log_10_ Observed *p*) of marker–trait associations for EV and precocity traits. SNP markers above the yellow horizontal lines (FDR = 0.05) were significantly associated with the trait. Markers in unknown regions are shown on chromosome 15, unanchored scaffolds on chromosome 16, and unassigned markers on chromosome 0.

The LDKNNi imputation in TASSEL imputed the genotypic data with 89.7% accuracy, masking a random 1% of markers. Quantile-quantile (QQ) plots demonstrated that the linear mixed model (LMM) effectively accounted for population structure, as the expected and observed *P*-values aligned closely without early deviations (Fig. 1). On the basis of a threshold of *P* < 1e-04, four single nucleotide polymorphism markers (SNPs) were associated with HT, nine with CW, four with CV, eight with TNM, and six with CNM (Supplementary Table 3). After adjusting for false discovery rate (FDR < 0.05), the number of significant SNPs decreased to two for HT, five for CW, four for CV, two for TNM, and five for CNM, explaining 11%–31% of the phenotypic variance for the associated traits (Fig. 2; Table 4). Among the significant SNPs, *Mint10079* was associated with HT, CV, TNM, and CNM, suggesting pleiotropic effects. Similarly, *Mint4004* was significantly associated with both HT and CNM, while *Mint11317* and *Mint9112* showed overlap in associations with CV/CW and TNM/CNM, respectively.

**Table 4:** Summary of significant SNPs associated with EV and precocity traits identified from GWAS

Trait	SNP ID	SNP position (bp)	FDR	SNP	MAF	Ma/Mi allele	R^2^ (%)
CV	*Mint11317*	Chr 2: 4672424	0.049	C/A	0.309	C/A	17
	*Mint10079*	Chr 3: 5338694	0.045	C/A	0.007	A/C	11
	*Mint7820*	Unknown	0.045	A/G	0.179	G/A	15
HT	*Mint4004*	Chr 5: 22548014	0.044	C/G	0.495	G/C	18
	*Mint10079*	Chr 3: 5338694	0.009	C/A	0.007	A/C	13
CW	*Mint11317*	Chr 2: 4672424	0.0004	C/A	0.309	C/A	31
	*Mint7123*	Chr 5: 10381062	0.028	T/C	0.037	T/C	12
	*Mint6881*	Chr 9: 27124204	0.040	A/G	0.076	A/G	11
	*Mint433*	Unknown	0.040	G/T	0.213	T/G	14
	*Mint8813*	Unknown	0.003	A/G	0.036	A/G	15
TNM	*Mint10079*	Chr 3: 5338694	0.038	C/A	0.007	A/C	11
	*Mint9112*	Chr 4: 18401039	0.023	C/G	0.069	G/C	23
CNM	*Mint4004*	Chr 5: 22548014	0.007	C/G	0.495	G/C	20
	*Mint10079*	Chr 3: 5338694	0.002	C/A	0.007	A/C	15
	*Mint1617*	Chr 1: 26883847	0.007	C/T	0.033	C/T	13
	*Mint9112*	Chr 4: 18401039	0.049	C/G	0.069	G/C	18
	*Mint3645*	Unknown	0.010	G/A	0.212	A/G	12

Ma, major allele; Mi, minor allele.

**Figure 3:**
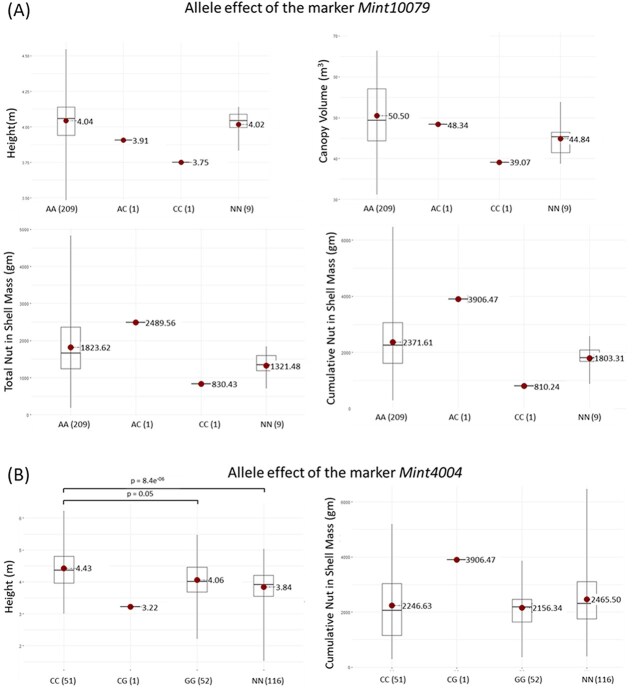
Allele effect of pleiotropic markers (A) *Mint10079* and (B) *Mint4004* associated with EV and precocity traits. Genotypic states of each marker are shown below the respective boxplots, with the numbers in parentheses indicating the number of accessions for each genotype. Mean trait values for each genotypic state are displayed next to the red dots, and significant differences (*P* < 0.05) are indicated above the corresponding boxplots. NN represents accessions lacking the significant SNP.

**Figure 4:**
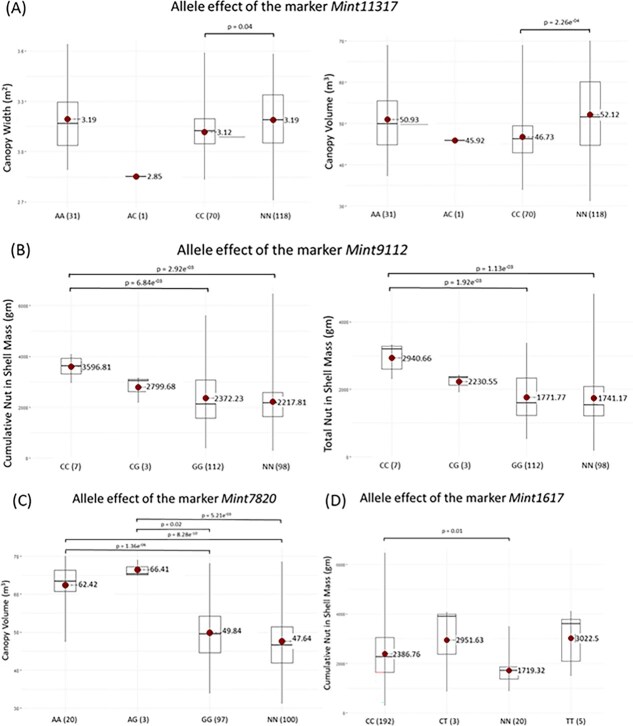
Allele effects of significant markers (A) *Mint11317* and (B) *Min9112* (C) *Mint7820* and (D) *Mint1617* associated with EV and precocity traits. Genotypic states of each marker are shown below the respective boxplots, with the numbers in parentheses indicating the number of accessions for each genotype. Mean trait values for each genotypic state are displayed next to the red dots, and significant differences (*P* < 0.05) are indicated above the corresponding boxplots. NN represents accessions lacking the significant SNP.

The effects of different allelic states (homozygous reference, homozygous alternate, heterozygous) of significant SNPs were analysed and compared with genotypes lacking that particular SNP (NN). For *Mint10079*, the homozygous alternate allele (CC) had a highly negative effect on both EV and precocity traits, though these differences were not statistically significant (*P* > 0.05) due to rarity (present in only one out of 220 accessions) (Fig. 3A). The heterozygous state (AC) exhibited a highly positive effect on precocity but was also a rare variant. Similarly, *Mint4004* exhibited significant positive effects for CNM (*P* < 0.001 for CC vs NN and *P* = 0.05 for GG vs NN) but had negative effects for HT when in the heterozygous state (CG, *n* = 1) (Fig. 3B). The heterozygote CG of *Mint4004* also had a highly positive effect on CNM although the difference was not significant due to the rarity of the allele. Other notable SNPs included *Mint11317*, which negatively affected CW and CV, with significant differences between CC and NN (*P* < 0.001) (Fig. 4A), and *Mint9112*, which positively influenced TNM and CNM, with CC showing the highest averages significantly different from GG (*P* < 0.001) and NN (*P* < 0.001) (Fig. 4B). Favourable SNPs such as *Mint7820* (for CV) (Fig. 4C) and *Mint1617* (for CNM) (Fig. 4D) also exhibited significant allele effects, while four markers associated with CW (*Mint8813, Mint7123, Mint6881*, *and Mint433*) and two markers associated with precocity (*Mint1617* and *Mint3645*) showed no significant allelic differences (data not shown).

Significant SNPs were mapped to seven of the 14 chromosomes in *M. integrifolia* (chromosomes 1, 2, 3, 4, 5, 7, and 9), while a few markers had unknown positions (Table 4). Genes closely associated with these significant SNPs (located within 5000 bp upstream and downstream of the SNP positions) were predominantly involved in regulating plant growth and development, pathogen defence, disease tolerance, and cell division (Table 5).

**Table 5:** Significant SNPs, closely associated genes, and their annotations from the latest whole-genome assembly of *M. integrifolia* along with probable gene functions in plant species

Traits	SNP ID	Closely associated genes	Gene annotation	Probable function/s	References
CV, CW	*Mint11317*	LOC122072123	Probable carotenoid cleavage dioxygenase 4	Controls lateral branching	[35]
CV, HT, TNM, CNM	*Mint10079*	LOC122073194	Glucan endo-1,3-beta-D-glucosidase-like	Cell division, pathogen defence, and pollen development	[36, 37]
CV	*Mint7820*	LOC122089925	1-Aminocyclopropane-1-carboxylate oxidase homolog 4-like	Regulates ethylene production affecting plant growth, development, and survival	[38]
HT, CNM	*Mint4004*	LOC122079354	Probable tRNA (guanine(26)-N(2))-dimethyl transferase 2	tRNA modification, plant protein synthesis, growth, development, and flowering time regulation.	[39, 40]
CW	*Mint7123*	LOC122079597	Transcription initiation factor TFIID subunit 15	Regulates gene expression and transcription pathways	[41]
CW	*Mint6881*	LOC122088113	DNA-directed RNA polymerase V subunit 5A-like	RNA production and reproductive development	[42]
CW	*Mint433*	LOC122074227	L-type lectin-domain containing receptor kinase IV.1-like	Bacterial pathogen defence, positive regulator of plant height	[43, 44]
CW	*Mint8813*	LOC122094272	Uncharacterized		
CNM, TNM	*Mint9112*	LOC122076299	Probable disease resistance RPP8-like protein 2	Pathogen resistance	[45]
CNM	*Mint1617*	LOC122074502	Uncharacterized		
CNM	*Mint3645*				

## Discussion

Large plant size and a lengthy life cycle, particularly an extended juvenile phase, pose significant challenges to the cost and time efficiency of fruit and nut breeding programmes. GWAS provides a powerful tool for overcoming these limitations by identifying genetic variants associated with key traits, facilitating MAS, and ultimately reducing selection cycles while increasing genetic gain. GWAS has successfully identified candidate trait-associated genes in several fruit and nut crops, including apple, pear, mango, kiwifruit, walnut, and almond ([46–49]; Pérez de los Cobos *et al*., 2023; [50]). The integration of GWAS with MAS has been shown to accelerate breeding cycles and improve genetic gain in tree fruit crops [51–53]. In macadamia breeding, GWAS has the potential to overcome traditional constraints by enabling early seedling preselection based on validated markers. This approach can significantly reduce costs and time by minimizing the number of trees required for phenotyping [3, 8]. EV and precocity are critical traits for macadamia selection, yet previous GWAS efforts for these traits have been limited. This study is the first to identify genetic links between EV and precocity traits in macadamia.

### Variability and heritability of traits in breeding population

Detection of a marker–trait association in GWAS is highly dependent on the extent of variation for the trait and a portion of this variation must be explained by genetic effects [54]. In this study, the mean values of traits varied across sites, with the highest EV observed at the Dunoon site and the lowest at Newrybar. Similarly, precocity had the highest mean value at the Bundaberg site and the lowest at Baffle Creek_S1. These differences in raw phenotypes suggest that growing location strongly influences EV and precocity traits in macadamia, a conclusion further supported by the high range of genetic variances across sites for these traits. Residual variances (V_e_) for EV traits were higher than additive genetic variances (V_a_) at all sites, indicating a large proportion of unexplained variation. The lack of replicates in the study prevented the estimation of nonadditive genetic variances, which may have contributed to the unexplained variance. In contrast, precocity traits exhibited higher V_a_ than V_e_ at most sites, suggesting stronger genetic control. The variation identified in this breeding population underscores its suitability for GWAS.

Heritability estimates for EV traits in this study demonstrate the complexity of these traits, with low to moderate H^2^ and h^2^ values. For instance, traits like CV (H^2^ = 0.23, h^2^ = 0.12) and CW (H^2^ = 0.21, h^2^ = 0.09) showed substantial environmental influence, suggesting that these traits may be challenging to improve genetically. The low heritabilities observed for these traits indicate that a significant portion of their expression is determined by environmental factors, making selection for these traits less effective unless extensive testing across different environments is conducted. In comparison, higher heritability estimates for vigour traits in other tree crops, such as cashew (H^2^ > 0.58, h^2^ > 0.51 for CW), walnut (h^2^ = 0.8 for HT), and pecan (H^2^ = 0.78 for HT) ([55–58]), suggest that these traits are more responsive to genetic selection in those species. This contrast underscores the importance of understanding species-specific genetic architectures. Similar to the finding in this study, Toft *et al*. [59] reported a very low H^2^ of 0.07 for CV of 4 to 5-year-old macadamia trees. Conversely, Hardner *et al*. [2] reported a higher heritability of CW at age five (H^2^ = 0.51–0.65 and h^2^ = 0.13–0.40). However, discrepancies between studies may stem from methodological differences, including the absence of pedigree information in earlier studies, which can result in underestimation of genetic effects [60]. In contrast, HT exhibited the highest heritability (H^2^ = 0.48, h^2^ = 0.27) among EV traits, reinforcing its potential as a practical target for breeding programmes aiming to enhance EV. This aligns with findings from macadamia and other tree crops like cashew and peach, where HT tends to show higher heritability compared to other vigour traits ([11, 55, 56, 59, 61]).

Precocity traits showed higher heritability than EV traits, suggesting a greater influence of genetic factors and reduced environmental variation. Comparable heritability estimates of early nut yield were reported in cashew (H^2^ = 0.38), kiwifruit (H^2^ = 0.35), and pecan (h^2^ = 0.35) [62–64]. In macadamia, similar estimates of TNM at age five (h^2^ = 0.19–0.37, H^2^ = 0.37–0.72) were reported by Hardner *et al*. (2019). These estimates contrasts with the low H^2^ of TNM (0.06) and CNM (0.13) reported by Hardner *et al*. [65] as well as TNM per tree (H^2^ = 0.13) by Toft *et al*. [59]. Differences in heritability estimates across studies underline the need for breeding programmes to carefully consider the population structure and selection strategies, as studies with selected progenies, such as that of Toft *et al*. [59] and Hardner *et al*. [65], may underestimate heritability due to reduced genetic variation [2]. These findings highlight the importance of accounting for environmental variation and leveraging appropriate statistical models, including those incorporating pedigree or genomic information, to more accurately estimate genetic parameters. Nonetheless, the higher genetic control over precocity traits makes them promising targets for genetic improvement, particularly in breeding programmes focused on early nut production.

### Correlation between EV and precocity traits

Positive correlations between EV and precocity have been previously established in tree crops including apple, pear, pecan, cocoa, and olive [17–20]. This study identified low to moderate phenotypic (r_p_) and genetic (correlations between BLUPs, r_g_) correlations between EV and precocity in macadamia, with the strongest associations observed for CW-TNM (r_p_ = 0.42; r_g_ = 0.29), followed by CV-TNM (r_p_ = 0.39; r_g_ = 0.28) and HT-TNM (r_p_ = 0.28; r_g_ = 0.26). Toft *et al*. [59], reported a higher r_g_ of 0.48 between early yield and CV. The correlations observed in this study were weaker than those found in other tree crops, such as walnut (r_p_ = 0.74) and cashew (r_p_ = 0.49) [66, 67]. The low to moderate r_g_ indicates that while EV can predict precocity to some extent, its utility as a selection criterion is limited. Selection strategies focused solely on increasing EV may result in marginal improvements in precocity, but the rate of genetic gain is likely to be slow.

The physiological significance of these correlations lies in the potential trade-off between early tree vigour and precocity. Increased EV may accelerate canopy development, allowing trees to intercept more light and accumulate carbohydrate reserves necessary for early flowering and fruiting. Rapid early development of above-ground biomass also ensures shading of the soil surface, reducing soil water evaporation and increasing the crop’s water use efficiency [68, 69]. However, if EV is strongly correlated with mature vigour, such selection for EV could have negative consequences, as high vigour at maturity is often associated with increased management costs and reduced yield efficiency [3]. Given these potential drawbacks, correlations between early-stage and mature vigour have been examined in other tree crops. Some studies have reported negligible correlations, providing opportunities to select early-bearing cultivars with low mature vigour [17, 70]. To optimize breeding strategies for macadamia, comprehensive data on vigour across developmental stages, particularly juvenile–mature correlations, is essential. Such information would enable the identification of genotypes that balance precocity and manageable vigour, optimizing both early performance and long-term productivity. Additionally, the observed correlations between EV and precocity may be influenced by hormonal signalling pathways. Brassinosteroids, in coordination with other plant growth hormones such as gibberellins and auxins, play a key role in regulating vegetative growth and flowering, particularly in the transition from juvenile to reproductive phases [71]. Their involvement in cell elongation, carbohydrate metabolism, and meristem activity suggests a potential molecular basis for the observed relationship between EV and precocity. Further genomic and physiological studies are needed to elucidate the mechanisms governing these correlations, particularly in identifying candidate genes and pathways underlying EV and precocity in macadamia.

### Genetic data and marker–trait associations

The genetic data used in this study comprised 7401 SNP markers obtained after the imputation and filtering of an initial dataset of 12 732 SNPs. Missing markers were imputed with high accuracy before applying quality control measures, including a call rate >50% and a minor allele frequency (MAF) >0.005. This contrasts with previous GWAS studies in macadamia, which filtered genetic data before imputation [32, 34], potentially reducing the number of usable markers. Pre-imputation filtering can impair imputation quality, as it may exclude markers that would otherwise meet quality thresholds after imputation [72].

A conventional GWAS approach filters SNPs with a MAF >0.05 to improve detection power, as rare alleles generally lack statistical power [73]. However, recent studies have extended this threshold, including markers with MAF as low as 0.03 [50], and even 0.01 [74], arguing that rare alleles, often arising from purifying selection, may carry causal variants critical for trait variation. Reflecting this, this study included markers with MAF >0.005, leading to the discovery of a significant SNP (*Mint11079*) with a very low MAF (0.007) associated with most traits under investigation. Although other significant SNPs had MAFs >0.03, this highlights the value of retaining low-frequency markers to capture rare but potentially important alleles. The low MAF of significant markers could shift with sample size, potentially affecting the proportion of explained variance [34].

Previous GWAS in macadamia excluded markers with MAF <0.025, although they identified many significant markers with MAF below this threshold due to a pre-imputation filtering of the SNP data [32, 34]. Excluding low MAF SNPs could prevent the identification of rare causal variants, as demonstrated in other crops: the *Brachytic2* locus (MAF <0.01) associated with plant height in maize and the *GS3* locus (MAF <0.01) linked to yield traits in rice [75, 76]. While rare alleles offer opportunities for genetic improvement, their utility in MAS must be approached cautiously. Statistical models designed for rare variant analysis in human genetics, such as burden tests or SKAT, may provide a useful framework for plants [77]. In the absence of such models for plant species, increasing marker density remains a viable strategy to enhance detection power [54]. Nevertheless, postvalidation, these rare alleles could be particularly valuable for gene editing approaches, such as target site mutation and CRISPR-Cas systems [78], to directly modify key loci associated with EV and precocity traits, enabling rapid genetic improvement and precision breeding in macadamia.

Despite the advantages of GWAS, false-positive associations remain a concern due to factors such as population structure, low-frequency causal alleles, and the multiple-testing burden [79]. The number of SNPs used in this study (7401) was relatively smaller compared to recent GWAS in other fruit and nut crops such as mango (100 000), apple (43 000), kiwifruit (9 million), walnut (364 275), and almond (60 581), which have leveraged higher SNP densities for improved detection power ([47, 49][80, 81]; Pérez de los Cobos *et al*., 2023; Y. Wang *et al*., 2024). Similarly, studies in grain crops typically employ larger SNP datasets, facilitating the identification of a greater number of significant associations [82–84]. Although this study utilized highly informative markers with stringent quality control, increasing SNP density in future studies could enhance genome-wide coverage and improve the detection of additional alleles linked to the traits of interest. While the SNP dataset in this study is not as extensive, it serves as a preliminary effort in applying genomic approaches to macadamia breeding. These findings provide an essential foundation for future research and breeding strategies, demonstrating the potential of GWAS to identify key genetic factors influencing important traits in macadamia.

Although this study effectively accounted for population structure and kinship using an LMM, as evidenced by QQ plots showing no early deviation from the null expectation, low MAF of a few SNPs is still a concern. Each marker–trait association must be validated before incorporating into MAS. Two common approaches are (1) independent validation in larger populations and (2) functional validation via transcriptomics, transgenesis, gene editing, or gene silencing [79]. While cross-validation is labour- and time-intensive, functional validation, particularly expression analysis of candidate genes, offers a more direct and efficient pathway to confirm GWAS results [85]. The step of validation through transcriptomics therefore provides better confidence in utilizing candidate genes/markers in MAS. By providing functional evidence, transcriptomics strengthens confidence in utilizing candidate genes and markers for MAS, bridging the gap between association studies and practical breeding applications.

### Allelic effects on phenotypic variation

Significant SNPs identified through GWAS explained 11%–31% of phenotypic variance, suggesting the presence of major QTLs controlling these traits. SNPs explaining >10% variance are considered effective for breeding applications [86]. Higher variance explanation also indicates stronger marker–trait correlations [87].

Visualizing the phenotypic effects of SNP alleles provides insights for MAS in breeding programme. In this study, favourable and unfavourable alleles were determined based on the phenotypic differences among different allelic states of each marker. For *Mint10079*, the rare allele AC (*n* = 1) showed a favourable effect on precocity with negligible impact on EV, while the rare CC allele (*n* = 1) reduced both EV and precocity, though these effects lacked statistical significance due to their rarity. Selecting for the AC allele, as such, could enhance precocity without compromising EV. Similarly, at another pleiotropic locus, *Mint4004*, the homozygous CC allele was favourable for HT, and the rare heterozygous CG allele positively influenced precocity while reducing EV. Selecting accessions for this rare heterozygous allele might be beneficial in identifying cultivars suitable for high-density planting systems, promoting small tree size and high precocity. Given the known importance of EV in stress tolerance and root growth [15, 88], further investigation into the effect of this SNP allele, especially on yield stability until maturity, is warranted. Considering that the favourable alleles in these instances are rare, thorough further investigation, as previously emphasized, is imperative.

For EV, individuals with homozygous CC allele at *Mint11317* displayed unfavourable effects, whereas those with AA or AG at *Mint7820* exhibited favourable impacts. Similarly, homozygous CC at *Mint9112* and *Mint1617* demonstrated highly favourable effects on precocity. Implementing MAS based on these findings involves genotyping progenies at the seedling stage, followed by selecting the most favourable alleles of these markers, postvalidation. This approach streamlines the selection process for improved EV and precocity, eliminating the need for manual phenotyping and population evaluation.

### Partially shared genetic mechanism between EV and precocity

The observed correlations between EV and precocity may be driven by shared molecular pathways controlling these traits. Pleiotropy, where a single gene affects multiple phenotypic traits, is a common mechanism underlying such correlations [46, 89, 90]. This study identified two SNPs, *Mint10079* and *Mint4004,* significantly associated with both EV and precocity. *Mint10079* was linked to HT, CV, TNM, and CNM, while *Mint4004* was associated with HT and CNM. Although the genetic correlation between EV and precocity traits was modest, the identification of shared loci highlights the potential for genetic co-regulation.

Pleiotropic genes influencing vegetative and reproductive development have been identified in other perennial crops. For example, *FLOWERING LOCUS T* (*FT*) is known to coordinate vegetative growth and flowering time in poplar, integrating environmental signals to regulate growth transitions [91, 92]. Similarly, significant loci associated with precocity have been linked to EV in pistachio [93]. Interestingly, pleiotropic loci have been observed even in cases where traits lack significant genetic correlations, as seen in wheat, where vigour and yield-related traits shared common genetic loci [94]. This suggests that pleiotropy may play a role in coordinating traits through shared pathways or regulatory mechanisms. From a breeding perspective, SNP markers with pleiotropic effects on EV and precocity enable simultaneous selection for favourable alleles across traits, enhancing MAS. However, thorough validation through independent testing and functional analyses, as mentioned before, is essential to ensure their reliability and practical application.

Earlier studies have shown that functional genes are often located near or within the regions of associated SNPs [95, 96]. With the availability of a well-annotated *M. integrifolia* genome [95], candidate genes were defined as those within 5000 bp of the associated SNPs. Among the eight putative candidate genes identified, two were found to exhibit pleiotropic effects, being associated with both EV and precocity traits: one encoding *glucan endo-1,3-β-D-glucosidase-like* and the other encoding *probable tRNA (guanine(26)-N(2))-dimethyl transferase 2*.

The *glucan endo-1,3-β-D-glucosidase-like* gene, officially classified as endo β-1,3-glucanase (β-1,3-G) [97], plays a critical role in various physiological and developmental processes in plants. *β-1,3-G* enzymes facilitate callose degradation at plasmodesmata, influencing cell-to-cell communication, axillary bud outgrowth, and shoot meristem differentiation [98, 99]. This function is critical for canopy expansion and biomass accumulation, which contribute to EV. Additionally, *β-1,3-G* has been linked to stomatal development [99], potentially enhancing gas exchange efficiency and supporting rapid growth. Beyond its role in vegetative processes, *β-1,3-G* is also implicated in floral induction by regulating callose turnover in meristematic tissues, thereby facilitating the transition from vegetative to reproductive growth [98]. The involvement of this gene in macadamia suggests its role in promoting vegetative growth while also enabling the transition to reproductive development.

The *probable tRNA (guanine(26)-N(2))-dimethyl transferase 2* gene is a key post-transcriptional regulator that stabilizes tRNA, enhances the accuracy of codon–anticodon pairing, and ensures proper tRNA folding under high temperature or oxidative stress [39]. tRNA modifications can enhance translational accuracy and efficiency, which are critical for optimal protein synthesis in plants [39]. This optimization of translation efficiency reduces energy wastage, potentially allowing plants to allocate resources more effectively to growth and defence. In *Arabidopsis*, mutants of this gene exhibited slower growth, reduced biomass, and delayed flowering, emphasizing its role in developmental regulation [40]. The association of this gene with both EV and precocity in macadamia suggests that enhanced protein synthesis efficiency may support robust early vegetative growth while ensuring timely floral transition. Efficient translation could facilitate the rapid accumulation of structural and regulatory proteins required for canopy expansion while simultaneously promoting the timely onset of reproductive development.

Other candidate genes associated with EV traits primarily regulate plant growth, reproductive development, pathogen defence, and transcriptional pathways in plant species. A *probable carotenoid cleavage dioxygenase* (*CCD)* gene associated with CV and CW showed a negative effect on these traits. *CCD*s regulate the synthesis of apocarotenoid hormones, which control lateral shoot growth and branching [100]. Reduced branching and compact growth due to *CCD* activity may influence overall tree architecture in macadamia, potentially affecting light interception and biomass allocation. Another candidate gene, *1-aminocyclopropane-1-carboxylate oxidase homolog 4-like* (*ACO*), linked to CV, is involved in ethylene synthesis [101]. The positive effect of *ACO* on EV traits in this study may be related to enhanced root development and improved cambial meristem activity, as ethylene signalling has been shown to regulate cell division and vascular differentiation [102]. Additionally, a locus annotated as *probable disease resistance RPP8-like protein 2*, known for its involvement in pathogen defence in *Arabidopsis,* was associated with precocity in this study [45]. While the precise function of this gene in precocity and yield remains unclear, enhanced pathogen resistance may reduce stress-induced delays in flowering and fruiting, thereby promoting early reproductive development. Future work should focus on functional validation of these candidate genes to confirm their roles and explore their regulatory networks in macadamia. Identified candidate genes, particularly the pleiotropic genes, are particularly promising.

## Conclusions

Macadamia is an ideal candidate for genomic-assisted breeding due to their long juvenile period, large size, and the costs associated with phenotyping, evaluation, and management. Despite these challenges, the application of MAS in macadamia breeding programmes has only recently gained attention. The present study, being the first to explore common genetic link between EV and precocity, thus has important implications for macadamia breeding. The findings offer significant insights into the variability of these traits in a diverse breeding population across different sites, emphasizing the influence of growing locations. The heritability estimates suggest that traits related to precocity are more heritable than EV. Marker–trait associations identified eight SNPs significantly associated with EV and five SNPs with precocity, including two pleiotropic SNPs linked with both traits. These pleiotropic markers present a unique opportunity for MAS in breeding, enabling the simultaneous enhancement of multiple desirable traits. Putative candidate genes associated with these markers, shedding light on their potential roles in physiological and developmental processes were investigated. Genes related to β-1,3-glucanases and guanine-methyl transferase influenced both EV and precocity traits. Additional candidate genes involved in plant growth, reproductive development, and defence mechanisms were also identified. The study also acknowledges the common challenges associated with GWAS, including false-positive associations, population structure concerns, and the importance of validating associations, particularly with low MAF markers. Overall, this study enhances our understanding of the genetic mechanisms underlying EV and precocity in macadamia, offering a pathway for the use of significant markers in MAS to accelerate the selection process in macadamia breeding.

## Methodology

### Plant material and phenotypic data

This study involves a subset of seedling progenies from the Australian macadamia breeding programme’s B1.2 population [103]. The entire progeny population consisted of 2345 biparental progenies from 204 families planted between 2001 and 2003 across nine locations in southeast Queensland and northeast New South Wales [104]. The subset in this study includes 904 progenies from 111 families planted from 2001 to 2003 across six sites in southeast Queensland: Alloway, Amamoor, Baffle Creek, Bundaberg, Dunoon, and Newrybar. Amamoor and Baffle Creek had two separate trials planted in 2002 and 2003, which were treated as distinct sites and are denoted as Amamoor_S1, Amamoor_S2, Baffle Creek_S1, and Baffle Creek_S2, respectively. These six sites were selected for analysis because phenotypic data for early vigour and precocity at age five were available only for these sites, while the remaining three locations lacked phenotypic records for these traits. Trees were planted in single tree plots using an incomplete block design with replication of families. Detailed information on trial layouts, block structure, and site-specific management is provided in Hardner [103] and Supplementary Table 3. Spacing was set at 4 m within rows and 8 m between rows [104]. Standard horticultural practices, including pruning, fertilization, irrigation, and pest management, were applied to maintain tree health [105].

Historical data on growth and precocity traits were utilized for this study. EV traits were represented by HT, CW, and CV measured in 5-year-old trees. HT was measured from the ground to the tallest point of the tress, using a retractable measuring pole. CW was measured from one end of the canopy to the other along the rows. CV was derived from the measured traits using the formulae: $CV=\frac{4}{3}\pi \times{\left(\frac{CW}{2}\right)}^2\times HT.$

Precocity was estimated using TNM and CNM measured at age five. Traits were assessed on an individual tree basis, following methods described by O’Connor *et al*. [106].

### Phenotypic data analysis

LMMs were fitted for each trait across sites using the *asreml* function in the ASREML-R package [107] in R v 4.3.2 [108]. Structured variance models were used to estimate genetic and residual variance components for each trait across sites, allowing for heterogeneous variances across different sites. Site was fitted as a fixed effect, while individual genotype, family, block, row, and column effects were fitted as random effects. The additive genetic relationship matrix was calculated from the available pedigree information, and its inverse was computed in ASReml-R using the algorithm of Luo [109]. Residuals were modelled with site-specific structures to account for spatial variability or independence within each site. For sites with spatial correlations, a separable 2D (row and column) first-order autoregressive (AR1 × AR1) structure was applied, as described by Gilmour *et al*. [110] At other sites, residuals were treated as independent (ID) in one or both dimensions, depending on the experimental conditions and site-specific patterns.

The data can be represented by *y_pi(jklm)_*, denoting the observation for the *p^th^* genotype (*g*) in the *i^th^* site (*a*), with associated family (*f*), block (*b*), row (*r*), and column (*c*), and the model can be defined as:

*y_pi(jklm)_ = μ + a_i_ + g_pi_ + f_ij_ + b_ik_ + r_il_ + c_im_ + e_pi(jklm)_* Model (1)

where *μ* is the overall mean, *a_i_* is the fixed effect of the *i^th^* site, *g_p_* is the random additive effect of the *p^th^* genotype across years (with distribution *g ~ N(0,*  ${\sigma}_{ai}^2A$) where *A* is the known additive relationship matrix based on pedigree and ${\sigma}_{ai}^2$is the additive genetic variance across *i^th^* site), *f_ij_* is the random effect of *j^th^* family at *i^th^* site, *b_ik_* is the random effect of *k^th^* block at *i^th^* site, *r_il_* is the random effect of *l^th^* row at *i^th^* site, *c_im_* is the random effect of *m^th^* column (tree position within rows) at *i^th^* site and *e_pi(jklm)_* is the residual error e *~ N (0, E_i_)* where *E_i_* is a block diagonal matrix specific to site i, modelled with residual variance–covariance structures as follows: *E =*  ${\sigma}_{ei}^2{\varSigma}_{ri}\bigotimes{\varSigma}_{ci}$ for Amamoor_S1, Amamoor_S2, Baffle Creek_S2, and Bundaberg,; *E*= ${\sigma}_{ei}^2I\bigotimes{\varSigma}_{ci}$ for Alloway; and *E*= ${\sigma}_{ei}^2I$ for Baffle Creek_S1 and Newrybar, where ${\varSigma}_{ri}$ and ${\varSigma}_{ci}$ are first-order autoregressive (ar1) spatial correlation matrices in the row and column dimensions, respectively, and *I* is the identity matrix. Wald tests of fixed effects were performed to identify the level of significance of differences among sites.

The variance components from Model 1 were used to estimate the narrow and broad sense heritabilities of each trait for each site using the following equations [111]:

$$h^{2}=\frac{\sigma_a^2}{\sigma_a^2+{\sigma}_f^2+{\sigma}_e^2\ }$$

$$H^{2}=\frac{\sigma_a^2+{\sigma}_f^2}{\sigma_a^2+{\sigma}_f^2+{\sigma}_e^2\ }$$

where ${\sigma}_a^2$ is the estimated additive genetic variance, ${\sigma}_f^2$ is the estimated family variance, and ${\sigma}_e^2$is the estimated residual variance. Variance components for each site used to estimate heritabilities are presented in Supplementary Table 2.

Predictions of total genetic effects (BLUPs) for each genotype and for each trait were made using an LMM based on Model 1 but with factor analytic (FA) genetic covariance models [112, 113]. In Model 1, the genetic effects across years were modelled using a diagonal structure, allowing for heterogeneous variances but assuming zero covariances between years [114]. The modified model (Model 2) replaced the diagonal structure with an FA model [113], which gives an approximation to an unstructured covariance matrix allowing for the direct estimation of genetic variances for each site and covariances between all pairs of sites.

All the phenotypic correlations and correlations between BLUPs presented in this study were calculated with the function *ggpairs* of the *GGally* package in R [115].

### Genotypic data analysis

#### Genotyping and SNP filtering

For the GWAS analysis, a subset of 220 genotypes was selected from the original 904 progenies. This subset was chosen based on yield performance of genotypes. Five high-yielding and five low-yielding progenies per family were selected [116]. These genotypes had previously been genotyped for 12 732 SNP markers by Diversity Array Technology (DArT) Pty Ltd. The markers used in this study were selected from an initial pool of ~1 million SNPs, which were assessed for reproducibility, and only those with >99% reproducibility were retained for analysis [117]. To address missing genotypes, the markers were imputed using the LD KNNi method in TASSEL v 5.2.89 [118]. Markers were filtered for various quality control measures including >50% call rate, >0.5% MAF, and 5% minimum heterozygosity. After filtering, 7401 SNP markers were retained for the analysis.

#### Association analysis

To adjust for the population stratification by accounting for family structure and cryptic relatedness [119], a principal component analysis (PCA) based on multidimensional scaling (MDS) of the genotypic dataset was performed in TASSEL. This was followed by obtaining the kinship (K) between genotypes, using the centred identity by state (centred-IBS) method as explained by Endelman and Jannink [120]. An LMM accounting for both of these population structure and relatedness (PCA + K model) was used to perform association analysis between the genotype and the phenotype in TASSEL [118]. The statistical method used for LMM is:

y = Xβ + Zu + e

where **y** is the vector of observations; β is an unknown vector containing fixed effects including genetic marker and population structure (Q); u is an unknown vector of random additive genetic effects from multiple background QTL for individuals or lines; X and Z are the known design matrices; and e is the unobserved vector of random residuals. The u and e vectors are assumed to be normally distributed with null mean and variance of $Var\ \left(\genfrac{}{}{0pt}{}{u}{e}\right)=\left(\genfrac{}{}{0pt}{}{G}{0\ }\ \genfrac{}{}{0pt}{}{0}{R}\right)$, where G = ${\sigma}_a^2K$ with ${\sigma}_a^2$ as the unknown additive genetic variance and *K* as the kinship matrix.

To visualize SNP markers associated with specific traits and verify that population structure was adequately accounted for, QQ and Manhattan plots were generated based on the association analysis. The QQ plot displayed the calculated SNP *P*-values on the y-axis against the expected uniform distribution of *P*-values on the x-axis, providing a visual assessment of deviations from the null hypothesis. The Manhattan plot highlighted markers significantly associated with the trait. The initial threshold for significant association was considered as *P* < 1e^−04^. To identify true-positive association of the markers and the phenotype, an FDR for each marker was calculated with the Benjamini and Hochberg approach [121, 122]. In this approach, all *P*-values from the GWAS were ranked in ascending order, with each *P*-value assigned a rank (*R*, e.g. 1, 2, 3, . . ., *K*, where *K* is the total number of markers). The FDR-adjusted *P*-value for each marker was calculated as: *P*-value (FDR) = $\frac{pR\ x\ 0.05}{K}$ [123], where *p*R refers to the rank of marker *P-*value and K is the total number of markers. The FDR-adjusted *P*-values were computed using the *p.adjust* function in R [124], and SNPs with FDR <0.05 were considered statistically significant. This threshold is widely used in plant GWAS studies aiming to identify candidate loci for further genetic and molecular analyses [125]. The allele effects of each significant SNP on the phenotype were visualized using the *ggbetweenstats* function from the *ggstasplot* package in R [126]. Pairwise tests were conducted to identify the level of significance between the different alleles.

#### Candidate gene detection

Putative candidate genes were identified using the latest whole-genome sequence of *M. integrifolia* [95] available on the NCBI database (www.ncbi.nlm.nih.gov/datasets/genome/?taxon=60698). Since the locations of most SNP markers were known, candidate genes within 5000 bp upstream and downstream of the SNP positions were detected. For SNPs with unknown positions, the allele sequences of these SNPs were blasted (https://blast.ncbi.nlm.nih.gov/Blast.cgi) against the NCBI nucleotide database for macadamia (SCU_Mint_v3 GenBank assembly [GCF_013358625.1]) to determine putative genes associated with the markers.

## Consent for publication

All authors approve the manuscript and consent to publication of the work.

## Acknowledgement

This work has been supported by the National Macadamia Breeding and Evaluation Program (MC19000, MC14000), funded by Hort Innovation Australia, using the Macadamia research and development levy and contributions from the Australian Government. Hort Innovation is the grower-owned, not-for-profit research and development corporation for Australian horticulture. Research funding was also provided by Queensland Government-supported Advance Queensland Industry Research Mid-Career Fellowship grant (AQIRF073-2022RD5; RM 2022002724). The funding bodies played no role in the design of the study and collection, analysis, and interpretation of data and in writing the manuscript. The funders had no role in the conceptualization, design, data collection, analysis, decision to publish, or preparation of the manuscript. The University of Queensland provided a higher degree research scholarship to P.D.P.

## Supplementary Material

Web_Material_uhaf162

## Data Availability

The datasets used and/or analysed during the current study are available from the corresponding author/s on reasonable request.
